# Investigation of the Effect of the Degree of Processing of Radix *Rehmanniae* Preparata (Shu Dihuang) on Shu Dihuangtan Carbonization Preparation Technology

**DOI:** 10.3390/molecules22071193

**Published:** 2017-07-18

**Authors:** Xianglong Meng, Meijing He, Rui Guo, Rui Duan, Fengxian Huo, Chenzi Lv, Bo Wang, Shuosheng Zhang

**Affiliations:** 1 Department of Herbology, College of Korean Medicine, Dongguk University, 38066 Gyeongju, Korea; sszywzh@126.com; 2 Institute of Pharmaceutical & Food Engineering and College of Chinese Materia Medica, Shanxi University of Chinese Medicine, Jinzhong 030619, Shanxi, China; hmjsxzyxy@163.com (M.H.); danil66@163.com (R.G.); 17612297661@163.com (R.D.); 18435166186@163.com (F.H.); lcz18435166779@163.com (C.L.); wb2399857464@163.com (B.W.)

**Keywords:** Shu Dihuang, Shu Dihuangtan, degree of processing, processing adjuvants, HPLC fingerprints, pyrolysis characteristics, Fourier transform infrared spectrometry (FTIR)

## Abstract

Carbonization of Radix *Rehmanniae* Preparata (Shu Dihuangtan) via stir-frying could increase its homeostasis maintaining and antidiarrheal effects. To ensure these pharmacological functions, the quality of the raw material (processed Rehmanniae Radix) must be well controlled. Therefore, we analyzed the effects of different degrees of processing and adjuvants on processed Rehmanniae Radix (Shu Dihuang) by High Performance Liquid Chromatography (HPLC) chromatographic fingerprints, thermal gravimetric analysis and Fourier transform infrared spectroscopy (FTIR). Based on the results from HPLC fingerprints combined with similarity analysis (SA) and hierarchical cluster analysis (HCA) the optimum processing method for Shu Dihuang was five cycles of steaming and polishing, which follows the ancient processing theory. The intensity of thermal weight loss rate peaked near 210.33 ± 4.32 °C or 211.33 ± 2.62 °C, which was an important indicator for the degree of processing of Shu Dihuang. A temperature near 290.89 ± 2.51 °C was the upper limit for carbonizing Shu Dihuangtan. FTIR spectroscopy analysis showed that the overall chemical composition of Shu Dihuangtan was affected by both the degree of processing and adjuvant, which are very important for its quality.

## 1. Introduction

Chinese medicinal herb preparation is an integral and important part of the Chinese medicine heritage, which has played an important role in the prevention and cure of diseases in China for thousands of years. Chinese herbal medicines need to be processed using different methods before being used, according to the state of an illness and the actual clinical need, in order to give full play to their disease preventive and curative effects, to overcome adverse reactions and guarantee their safety and effectiveness. 

Rehmanniae Radix (Dihuang in Chinese), which is derived from the root of *Rehmannia glutinosa* Libosch, has long been used in traditional Chinese medicine (TCM). It was first reported in the Canon on Medicinal Herbs by Divine Ploughman (ca. 200 B.C.) from Shen-nong Bencao Jing [1]. It is classified as a high-grade (very safe) medicine. In traditional Chinese medicine, based on the processing methods, there are two types of Rehmanniae Radix that are frequently used in clinical prescriptions, raw Rehmanniae Radix (Sheng Dihuang) and Radix Rehmanniae Preparata (Shu Dihuang). Sheng Dihuang is obtained by drying the fresh root of *R. glutinosa*. It is known to be able to “reduce the heat in blood, nourish yin and promote the production of body fluids”, and thereby is used for treating maculation, nosebleeds, rash, and skin eruptions. In contrast, Shu Dihuang is prepared by steaming or braising the raw Rehmanniae Radix with yellow rice wine (or not). It can “nourish yin and replenish blood, thus reinforcing essence and marrow”, and is thus used for treating anemia, diabetes, dizziness, tinnitus, nocturnal emission and palpitations [2,3]. The preparation method of Shu Dihuang is very controversial because of the inconsistency between different heating methods (steaming or braising, cycles and time) and processing adjuvants (yellow rice wine or none). The old approach, “steaming and drying for several cycles (generally nine times)”, has been regarded as the golden standard method according to the ancient processing theory. 

Carbonization by stir-frying is a common and important method for processing traditional Chinese medicinal materials, and the herbs’ homeostasis maintaining and antidiarrheal effects are more remarkable after being processed in this way [4]. The carbonization of Radix Rehmanniae Preparata (Shu Dihuangtan) is accomplished through stir-frying or calcining. However, there’s no consistent conclusion on which processing technology works the best, and there are also no related reports comparing different processing methods and their effects on Shu Dihuangtan’s pharmacological actions. This lack of comparative studies has hindered the optimization of processing technology and the improvement of Shu Dihuangtan quality. 

Thermal analysis [5] is a groups of techniques used to measure the physical properties of materials as they change with temperature and time when subjected to a programmed heating process. It includes thermal gravimetry (TG), derivative thermogravimetry (DTG), differential thermal analysis (DTA), thermo-mechanical analysis (TMA), dynamic mechanical analysis (DMA), and combined use of thermal gravimetry with mass spectrometry (MS), Fourier transform infrared spectroscopy (FTIR), or gas chromatography (GC). In recent years, these technologies have been progressively applied to traditional Chinese medicine studies, such as thermal analysis of chemical compositions [6] and decomposition [7], identification of product origin [8] or relatives [9], etc. We have applied these techniques to streamline the processing of traditional Chinese medicines. Previously, we used TG/DTG methods to simulate the processing procedure of Zushima, in order to qualitatively demonstrate the overall compositional changes [10,11], and also analyzed the effects of pyrolysis in an inert Ar atmosphere on the preparation of gallic acid, rhubarb, mudan and burnet [12]. We also calculated the energy dynamics during the stir-frying process based on the data obtained from thermal analysis techniques [13]. These applications have greatly enhanced our experimental and theoretical understanding of the processing procedures of traditional Chinese medicines.

In this study, we compared the HPLC fingerprints of Shu Dihuang samples prepared with different degrees of processing and adjuvants, and classified the fingerprint data using several chemometrics methods, such as similarity analysis (SA) and hierarchical cluster analysis (HCA). According to the statistical results and chromatographic fingerprints, we identified the peaks corresponding to different degrees of processing and adjuvants, and analyzed how these peaks changed during the processing procedure. Moreover, the pyrolysis characteristics of Shu Dihuang with different degrees of processing and adjuvants were compared by thermal gravimetric analysis (TGA), and the upper and optimum temperature limit for carbonization were quantified. Finally, Fourier transform infrared (FTIR) spectroscopy was used to qualitatively analyze the discrepancies between different carbonization processes for Shu Dihuangtan at the optimum temperature. Overall, this study provides a scientific support for studying carbonization technology based on the ancient processing theory.

## 2. Results and Discussion

### 2.1. The Diverse HPLC Fingerprints of Shu Dihuang with Different Degrees of Processing and Adjuvants

HPLC chromatographic conditions were optimized based on references and actual experiments. The HPLC chromatograms of Shu Dihuang whole extract had unsteady baselines and the reproducibility was bad, which was mainly due to the complex chemical composition changing as a result of the different steaming and drying times and processing adjuvants. Also, there was no peak after 140 min and some peaks around 20 min were not completely separated. To simplify the HPLC chromatographic fingerprint and time frame, different parts of the extract were separated and the collection time was shortened. The petroleum ether, chloroform, ethyl acetate, *n*-butyl alcohol and water fractions extracted from test samples were assessed with HPLC chromatographic fingerprint in many preliminary experiments, and water fraction had the best reproducibility. Therefore, in this experiment we mainly used the water fraction for HPLC chromatographic fingerprinting. 

For the methodology validation, the RSD values calculated from the relative retention time and relative peak area were less than 3%, which demonstrated that the instrumental and sampling precision were good, and the extraction method and chromatographic conditions were appropriate.

**Figure 1:**
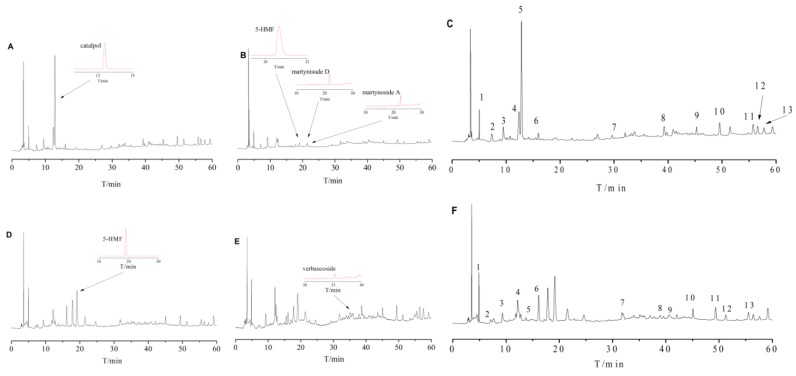
HPLC chromatograms of Shu Dihuang. Notes: (**A**–**C**) HPLC figures of Shu Dihuang (SW1, SW3 and SW3) determined at 205 nm; (**D**–**F**) HPLC figures of Shu Dihuang (SW6, S4 and SW6) determined at 284 nm.

**Figure 2:**
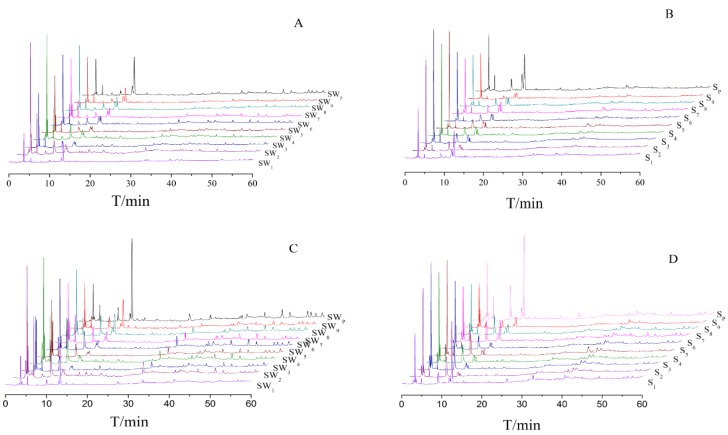
Overlaid HPLC chromatograms of Shu Dihuang. Notes: (**A**) and (**B**) were determined at 205 nm; (**C**) and (**D**) were determined at 285 nm; (**A**) and (**C**) belong to Shu Dihuang processed with yellow rice wine; (**B**) and (**D**) belong to Shu Dihuang.

**Figure 3:**
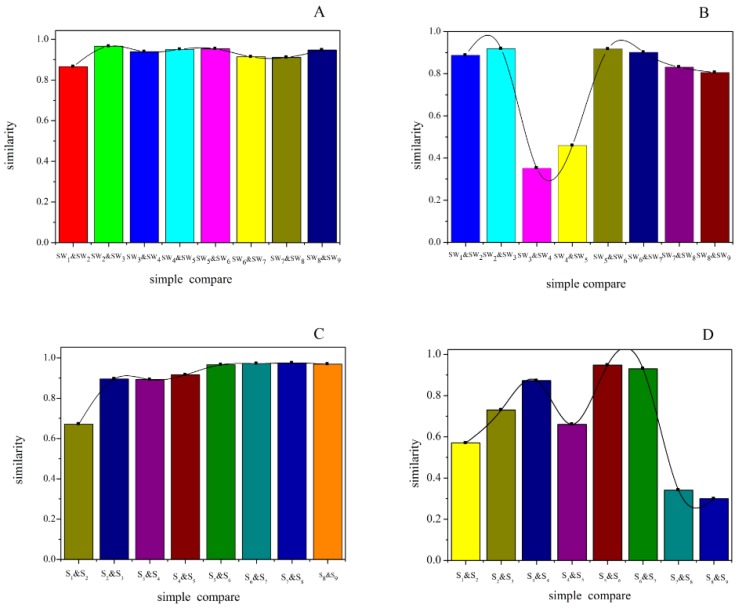
The relative similarity of Shu Dihuang. Notes: (**A**) and (**C**) were determined at 205 nm; (**B**) and (**D**) were determined at 285 nm; (**A**) and (**B**) belong to Shu Dihuang processed with yellow rice wine; (**C**) and (**D**) belong to Shu Dihuang.

With SP and SWP as references, 13 common peaks were determined at 205 nm and 284 nm detection wavelengths, as shown in Figure 1E,F. Because of the different processing procedures of Shu Dihuang, the HPLC chromatographic fingerprints were also very diverse. The overlaid HPLC chromatograms of Shu Dihuang are shown in Figure 2. Their similarities were shown in Table 1, Table 2, Table 3 and Table 4. With Shu Dihuang (SWP) as the reference (Table 3 and Table 4), the similarities were 0.442–0.969 and 0.472–0.949 at 205 and 284 nm detection wavelengths, respectively. When compared with the generated common peak HPLC chromatogram (R), SW3 had higher similarity values (0.972 and 0.949) at both 205 and 284 nm detection wavelength. For the relative similarity at 205 nm, there was higher similarity between SW2 and SW9 (greater than 0.9), and lower similarity between SW2 and SW1 (0.866) (Figure 3). SW4 and SW5 both had higher similarities with the other samples, and they also had relatively high similarity with SWP and R.

For the relative similarity at 284 nm, the two smallest values appeared between SW4 and SW3, and SW5 and SW4 (0.352 and 0.459, respectively), and other samples’ relative similarities were all greater than 0.8. As shown in Table 3, the similarities of SW5 with the other samples were higher than SW4. Compared with SWP and R, SW5 also had higher similarity. Overall, SW5 had higher similarity than SW4.

With Shu Dihuang (SP) as the reference (Table 4 and Table 5), similarities were 0.813–0.972 and 0.497–0.854 at 205 and 284 nm detection wavelengths, respectively. When compared with the generated common peak HPLC chromatogram (R), S5 (at 205 nm) and S3 (at 284 nm) had high similarities, which were 0.972 and 0.854. For the relative similarity at 205 nm, the smallest similarity appeared between S1 and S2 (0.671), and the other samples’ relative similarities were all greater than 0.9. S4 and S5 both had higher similarities with the other samples. But most of the other samples had higher similarities with S5. For the relative similarity at 284 nm, there were two small similarity values between S9 and S8 (0.3), and S8 and S7 (0.341). Similarly, S5 had higher similarity compared with the other samples.

**Figure 4:**
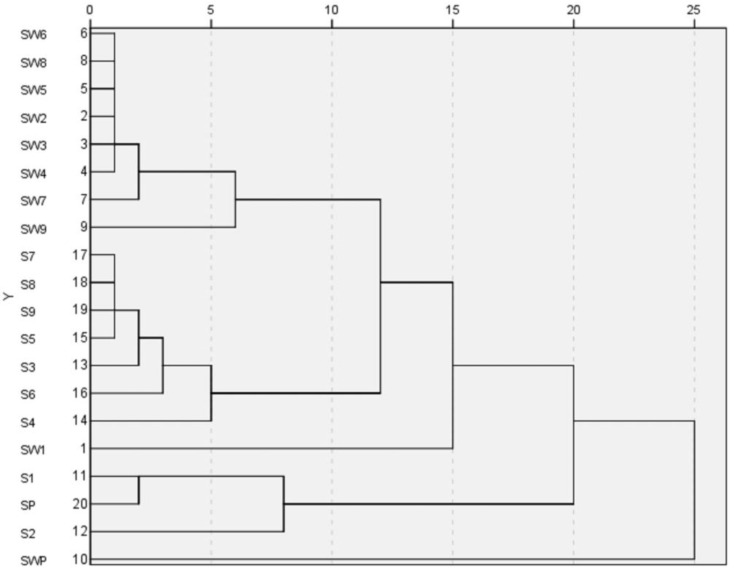
Dendrogram of the hierarchical cluster analysis of Shu Dihuang determined at 285 nm.

HCA is a way to structure a complicated set of observations into unique and mutually exclusive subject groups (clusters) that are similar to each other with respect to certain characteristics. The HCA results of S1~S9, SW1~SW9, SP and SWP of Shu Dihuang determined at 285 nm were shown in Figure 4. Overall, it was clear that Shu Dihuang and Shu Dihuang processed with yellow rice wine were clustered separately into two groups, indicating that the processing adjuvant (yellow rice wine) caused changes in the composition and/or content of components. This further reflected the differences seen in relative similarity. SW5 and most of the samples processed with yellow rice wine were clustered into the same group, including SW6, SW8, SW2, SW3, SW4, SW7 and SW9. Similarly, S5, S7, S8, S9, S3, S6 and S4 were clustered into another group. Moreover, this result was consistent with the previous results.

In summary, the overall chemical composition of Shu Dihuang was affected by steaming and polishing cycles and processing adjuvant (yellow rice wine). Catalpol, a kind of cycloalkene ether terpenoid, was detected at 205 nm, while 5-HMF and verbascoside were mainly detected at 284 nm. The related studies had shown that the content of cycloalkene ether terpenoids was reduced while the content of 5-HMF was increased as the degree of processing increased, and 5-HMF was an important indicator for the quality of Shu Dihuang [14,15]. Therefore, the correlation result at 285 nm detection wavelength was a critical parameter. This also led to the low number of common peaks (13) and the absence of reference substances in common peaks. Based on the results from comparing the effects of steaming and polishing times and processing adjuvants, we could draw the conclusion that the overall chemical composition experienced a great change from three to five cycles of steaming and drying, and five cycles was a simple processing method of Shu Dihuang which obeyed the ancient processing theory. These results also suggested that the degree of processing must be taken into account when preparing Shu Dihuangtan. 

### 2.2. The Pyrolysis and Combustion Characteristics of Shu Dihuang with Different Degrees of Processing and Adjuvants

**Figure 5:**
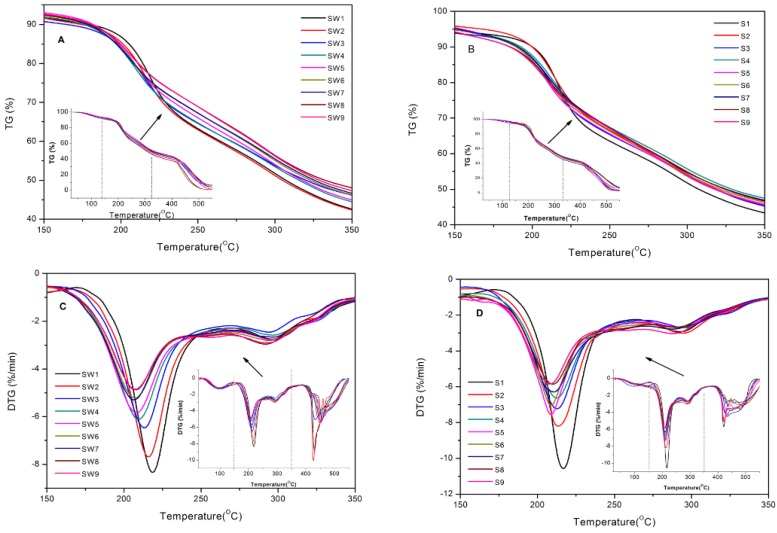
Pyrolysis and combustion TG/DTG curves for Shu Dihuang. Notes: (**A**) and (**C**) were respectively TG and DTG curves of Shu Dihuang processed with yellow rice wine; (**B**) and (**D**) were respectively TG and DTG curves of Shu Dihuang.

The TG-DTG curves of Shu Dihuang are shown in Figure 5, and the pyrolysis parameters and combustion characteristics are listed in Table 5. Three different pyrolysis and combustion stages were observed: drying and dehydration, pyrolysis and combustion, carbonization and combustion. Since the pyrolysis and combustion characteristics were used to simulate the preparation process of Shu Dihuangtan, we focused more on the pyrolysis and combustion stage.

In the pyrolysis and combustion stagef, two obvious thermal weight loss phases and two thermal weight loss rate peaks appeared for Shu Dihuang with different steaming and drying cycles and adjuvants. For Shu Dihuang processed with yellow rice wine, the initial temperature and the final temperature in these two phases were 151 ± 8.16 °C to 266.67 ± 1.56 °C and 266.67 ± 1.56 °C to 386.67 ± 4.32 °C, respectively. Moreover, the intensities of the two thermal weight loss rate peaks were 6.07 ± 1.16%·min^−1^ and 2.74 ± 0.15%·min^−1^, and they appeared at 210.33 ± 4.32 °C and 294.89 ± 1.66 °C, respectively. Moreover, the pyrolysis-combustion characteristics of Shu Dihuang processed with no adjuvant were similar. The initial temperature and final temperature in the two phases were 158.22 ± 6.75 °C to 266.33 ± 5.37 °C and 266.33 ± 5.37 °C to 378.56 ± 4.30 °C, respectively. The intensities of the thermal weight loss rate peaks were 7.25 ± 1.38%·min^−1^ and 2.80 ± 0.14%·min^−1^, and they appeared at 211.33 ± 2.62 °C and 290.89 ± 2.51 °C, respectively.

Along with the increase of steaming and polishing cycles, both the mean intensity of the two thermal weight loss rate peaks and the temperature interval (initial and final temperature) decreased in the pyrolysis and combustion stage. For Shu Dihuang processed with yellow rice wine, the initial and the final temperatures for SW2 were respectively continuous within the range of 163 °C and 396 °C, and the intensities of the thermal weight loss rate peaks were 7.68%·min^−1^ and 2.78%·min^−1^. The initial and the final temperatures for SW5 were between 144 °C and 385 °C, and the intensities of thermal weight loss rate peaks were 5.84%·min^−1^ and 2.77%·min^−1^. For SW8, the initial temperature was 145 °C and the final temperature was 381 °C, and the intensities of thermal weight loss rate peaks were 4.88%·min^−1^ and 2.96%·min^−1^. For Shu Dihuang processed without adjuvant, the temperature interval for S2 started at 163 °C and finished at 396 °C, and the intensities of thermal weight loss rate peaks were 8.20%·min^−1^ and 3.00%·min^−1^. The initial and final temperatures for S5 were continuously within 148 °C and 377 °C, and the intensities of thermal weight loss rate peaks were 7.55%·min^−1^ and 2.46%·min^−1^. For S8, the initial temperature was 158 °C and the final temperature was 373 °C, and the intensities of thermal weight loss rate peaks were 5.86%·min^−1^ and 2.68%·min^−1^. However, overall, the differences of thermal weight loss and thermal weight loss rate were very small from SW5 to SW9. Also, the peak temperature of thermal weight loss rate of SW5 was similar to SW6-SW9 (Figure 5), demonstrating that the trends for Shu Dihuang processed with or without adjuvant were similar, so we hold the opinion that five cycles represent a relatively good processing method.

For Shu Dihuang processed with the same steaming and polishing cycles, the pyrolysis-combustion characteristics were also different because of the processing adjuvants (with yellow rice wine or not). The intensity of the thermal weight loss rate peak was less for the Shu Dihuang processed with yellow rice wine compared to the Shu Dihuang processed without adjuvants at the same steaming and polishing cycle. Meanwhile, the Shu Dihuang processed with yellow rice wine spanned a smaller temperature interval during its thermal weight loss.

To summarize, the degree of processing and adjuvant are important conditions in the carbonization process for preparing Shu Dihuangtan. The intensity of thermal weight loss rate peak near 210.33 ± 4.32 °C or 211.33 ± 2.62 °C was an important indicator of the degree of processing of Shu Dihuang. Moreover, the corresponding temperature of the thermal weight loss rate peak in the second stage of pyrolysis-combustion near 290.89 ± 2.51 °C was the upper temperature limit of carbonization of Shu Dihuang. 

### 2.3 FTIR Diversity of Shu Dihuangtan

**Figure 6:**
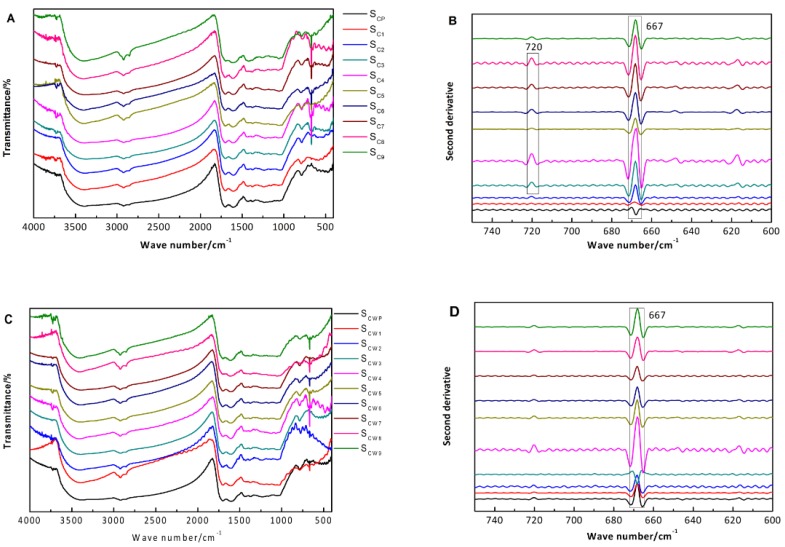
FTIR Spectra of Shu Dihuangtan. Notes: (**A**) and (**B**) were belong to Shu Dihuangtan, (**C**) and (**D**) were belong to Shu Dihuangtan processed with yellow rice wine.

As shown in Figure 6, there were nine common peaks in the FTIR spectra located at 3000, 2920, 1702, 1650, 1609, 1430, 785, 720 and 667 cm^−1^, respectively. The Shu Dihuangtan peaks of (CS1~CS9, CSW1-~CSW9, CSP and CSWP) at 3000 cm^−1^ were possibly due to the stretching vibration of ν_=C-H_ [16], and the peak near 1650 cm^−1^ was assigned to the stretching vibration of ν_=C-C_ [17]. Combining these data with the out-of-plane bending vibration at 720 cm^−1^ from the second derivative spectrum, we concluded that there was an olefin structure with a bis-substituted *cis* single olefin in Shu Dihuangtan [18]. Furthermore, the peak near 2920 cm^−1^ was classified as the stretching vibration of -CH_3_ and -CH_2_ [19], the peak at 1702 cm^−1^ belonged to the carboxyl C=O stretching vibration, and the peaks at 1609 and 1430 cm^−1^ were the characteristic absorption peaks of aromatic acidic material [20]. Combining these data with the aromatic compound C–H vibration absorption peak at 785 cm^−1^, we speculated that there was benzodiazepine material in Shu Dihuangtan [21]. In addition, the peak at 667 cm^−1^ was classified as the in-plane bending vibration of CO_2_ [22], and the peaks at 720 cm^−1^ and 667 cm^−1^ had significant differences in the second derivative spectrum. The peak intensities of CS5 and CSW5 were smaller than others, while the peak intensities of CS4 and CSW4 were much stronger.

**Figure 7:**
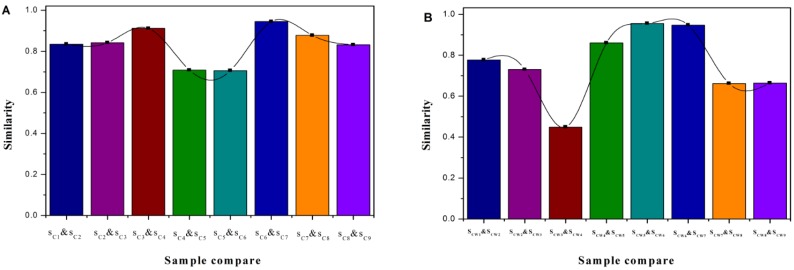
The FTIR relative similarity of Shu Dihuang. Notes: (**A**) was belong to Shu Dihuangtan, (**B**) was belong to Shu Dihuangtan processed with yellow rice wine.

With Shu Dihuangtan (CSP) as the reference (Table 6), the similarities ranged from 0.285 to 0.871 (CS1 to CS9, processed with no adjuvants). The highest similarity was between CS1 and CSP (0.871), while the lowest similarity was between CS4 and CSP (0.285). For the similarities between any two samples, CS3 and CS9 were relatively higher. Meanwhile, the relative similarity between CS3 and CSP as well as the sample with several cycles of steaming and drying (generally nine cycles) in the ancient theory were both obviously high. For the relative similarities among CS1-CS9, as shown in Figure 7, the highest two were CS4 and CS3 (0.912), and CS7 and CS6 (0.945); the lowest two were CS5 and CS4 (0.709), and CS6 and CS5 (0.706).

With Shu Dihuangtan (CSWP) as the reference (Table 7), the similarities ranged from 0.623 to 0.951 (CSW1 to CSW9, processed with yellow rice wine). The highest similarity was between CSW7 and CSWP (0.951), while the lowest similarity was between CSW8 and CSP (0.623). For the similarities between any two samples, CSW4 and CSW9 were relatively higher, and CSW5 and CSW6 had the highest similarity (0.955). For the relative similarities among CSW1-CSW9, as shown in Figure 6, the smallest was CSW4 and CSW3 (0.449), and the similarities among CSW4-CSW7 were kept at high levels.

Overall, all samples’ similarities were different and most of them were relatively small. The global chemical composition of Shu Dihuangtan was affected by both the degree of processing and adjuvant.

## 3. Materials and Methods

### 3.1. Materials and Reagents

Acetonitrile (chromatographic grade) was purchased from Beijing Bailingwei Tech Co., Ltd. (Beijing, China). Methanol (analytical reagent) was purchased from Tianjin Beichenfangzheng Reagent Factory (Tianjin, China). Phosphoric acid (chromatographic grade) was purchased from Tianjin Kemiou Chemical Development Center (Tianjin, China). Potassium bromide (spectroscopy grade) was purchased from ANPEL Laboratory Technologies (Shanghai, China). Standard reagents: acteoside, catalpol, 5-hydroxymethylfurfural, rehmaionoside A and rehmaionoside D (production batch numbers 150401, 150331, 160126, 140506 and 140402, respectively) were purchased from Chengdu Pufei De Biotech Co., Ltd. (Chengdu, China). The mass fraction of all standard reagents were ≥98%. The crude plant materials of raw Rehmanniae Radix (Sheng Dihuang) used in this study were collected from Xiangfen (Shanxi Province, China). They were confirmed as the roots of *Rehmannia glutinosa* Libosch. By Prof. Zhang Shuosheng from Shanxi University of Chinese Medicine of China.

### 3.2. Sample Preparation

#### 3.2.1. The Preparation of Different Types of Shu Dihuang

Sheng Dihuang was placed in a steamer and heated for 6 h. Then the samples were put into a drying oven and dried for 24 h at 60 °C. This steaming and polishing step was repeated nine times on nine samples which were separated after each cycle. Samples were cut in pieces, dried at 60 °C and labeled from S1 to S9 [23,24,25,26]. These were the sample batch processed without adjuvants.

For the sample batches processed with adjuvant, yellow rice wine was used as the processing adjuvant, and the ratio between the yellow wine and herb was 10:4. The other conditions were kept the same as above. The samples were labeled from SW1 to SW9.

According to Chinese National Pharmacopoeia (2015), Sheng Dihuang was steamed until the herb turned black. Then the samples were exposed to sunlight until 80% dry and cut into thick slices or pieces and dried completely. This sample was labeled as SP.

According to Chinese National Pharmacopoeia (2015), Sheng Dihuang was steamed with yellow rice wine (Tapai yellow rice wine from Zhejiang Tapai Shaoxing Wine Co., Ltd., Shaoxing, China) until the wine was fully absorbed. The sample was then exposed to sunlight until the surface was slightly dried. After that, the sample was cut into thick slices or pieces and dried completely. The sample was labeled as SWP (30~50 kg of wine was used in every 100 kg of Sheng Dihuang). 

To prepare carbonized Shu Dihuangtan samples a simultaneous thermal analyzer (STA-409C, NETZSCH, Waldkraiburg, Germany) was used to heat S1~S9, SW1~SW9, SP and SWP from room temperature to the maximum temperature indicated in Section 2.2 at the rate of 10 °C·min^−1^. The samples were marked as SC1~SC9, SCW1~SCW9, SCP and SCWP.

#### 3.2.2. Sample Preparation for HPLC

The tested samples were crushed into powder with a pulverizer for 1 min, and passed through a 40 μm-mesh sieve. A portion of each sample (1.0 g) was accurately weighed and placed into a 50 mL flask, added with methanol and soaked overnight (12 h) at room temperature. Then the sample was extracted with an ultrasonic extraction device for 1.5 h, cooled down to room temperature, the weight loss supplemented with fresh solvent, and filtered through filter paper. An aliquot (25 mL) of the filtrate was dried using a water-bath. The residue was dissolved with deionized water and extracted successively with petroleum ether, chloroform, ethyl acetate, and *n*-butyl alcohol. The aqueous solvent fraction was evaporated on a rotary vacuum evaporator at 60 °C until the weight of the dry extract was constant. Then the dry extract was dissolved with 20% methanol in a 10 mL flask and filtered through 0.45 μm microporous membrane. A 10 μL aliquot of the filtrate was then injected for fingerprint analysis. Reference solutions (standards) containing catalpa alcohol (202.4 μg·mL^−1^), rehmannioside D (78.2 μg·mL^−1^), rehmannioside A (83 μg·mL^−1^), acteoside (200 μg·mL^−1^), and 5-HMF (2276 μg·mL^−1^) were prepared with 20% methanol and filtered through 0.45 μm microporous membranes. A 10 μL aliquot of the filtrate was injected for fingerprint analysis.

### 3.3. Chromatographic Conditions, Methodology Validation and Data Analysis

All HPLC analyses were performed with a U3000 series system (Thermo Fisher Scientific, Waltham, MA, USA). A Hypersil GOLD aQ C18 column (250 mm × 4.6 mm, 5 μm) was used. The mobile phase consisted of 0.1% phosphoric acid (A) and acetonitrile (B) [27]. The gradient program was developed as shown in Table 8. The flow rate was kept at 1.0 mL·min^−1^ and the column temperature was maintained at 25 °C. The injection volume was 10 μL and the detection wavelengths were selected at 205 and 284 nm.

All the tests below were carried out on Shu Dihuang prepared as described in Section 3.2. The precision was determined by repeating HPLC injections of the same sample six times per day. Six independent samples (prepared as described in Section 3.2.2) were extracted and analyzed in parallel for the evaluation of reproducibility. The stability was assessed by measuring a single sample solution stored at room temperature at 0, 2, 4, 8, 16 and 24 h.

HPLC chromatographic data of all the samples were integrated automatically and exported as *.AIA format files for further processing. The *.AIA files were imported into the similarity evaluation system for chromatographic fingerprint of TCM (Version 2004 A, Committee for the Pharmacopeia of PR China, Beijing, China). A reference sample was selected stochastically from the most representative samples among the middle of the analytical sequence to generate a template. Then, all of the samples were overlaid based on the template, and common peaks were aligned to the ones in template. The relative retention time (RRT) and relative peak area (RPA) were calculated simultaneously and were exported as an excel file for further statistical analysis. Hierarchical cluster analysis (HCA) was performed by IBM SPSS 22.0 (International Business Machines Corporation, Armonk, NY, USA).

### 3.4. Thermal Gravimetric Analysis (TGA)

A simultaneous thermal analyzer (STA-409C, NETZSCH) was used for TGA. The herb sample (30 ± 5 mg) was placed in a crucible under a constant flow of simulated air (100 mL·min^−1^, N_2_:O_2_ = 4:1). The temperature of the crucible was increased from room temperature to 600 °C at 10 °C·min^−1^ (heating rate). The experiments were repeated twice.

### 3.5. Fourier Transformation Infrared Spectrometry (FTIR) Experiment

A Fourier transform infrared spectrometer (MB104, BOMEM, Quebec, Canada) was used for FTIR. The tests were performed on KBr pellets with a middle infrared detector, and the spectral resolution was 4 cm^−1^, the detection range was 4000~500 cm^−1^, and the scan signal was accumulated 32 times. Automatic correction, curve smoothing and longitudinal coordinate normalization were performed on the collected data with the Thermo Fisher Nicolet FTIR OMNIC 8.0 software.

## 4. Conclusions

In summary, the degree of processing (steaming and polishing times) and processing adjuvant (yellow rice wine) had a great influence on the overall chemical composition of Shu Dihuangtan. This influence also caused the differences in Shu Dihuang’s pyrolysis-combustion characteristics and the FTIR spectrogram similarities. 210.33 ± 4.32 °C can be used as optimum temperature reference. The optimum processing method of Shu Dihuang was steaming and polishing for five cycles, which followed the ancient processing theory. Since the quality of Shu Dihuang is closely related to the quality of Shu Dihuangtan, the degree of processing and use or not of processing adjuvant of Shu Dihuang are very important for Shu Dihuangtan preparation. As for the reason why the processed of Shu Dihuang is steaming and drying for several cycles (generally nine times) in the ancient theory, we need to further combine these results with the pharmacology of Shu Dihuangtan to confirm the reasons.

## Figures and Tables

**Table 1:** The similarity result of Shu Dihuang processed with yellow rice wine determined at 205 nm.

	SW1	SW2	SW3	SW4	SW5	SW6	SW7	SW8	SW9	SWP	R
SW1	1	0.866	0.773	0.675	0.65	0.611	0.485	0.542	0.486	0.735	0.754
SW2	0.866	1	0.966	0.884	0.86	0.848	0.745	0.807	0.769	0.562	0.934
SW3	0.773	0.966	1	0.939	0.923	0.917	0.846	0.893	0.868	0.438	0.972
SW4	0.675	0.884	0.939	1	0.951	0.932	0.896	0.913	0.904	0.359	0.965
SW5	0.65	0.86	0.923	0.951	1	0.953	0.919	0.923	0.94	0.323	0.969
SW6	0.611	0.848	0.917	0.932	0.953	1	0.914	0.941	0.925	0.278	0.954
SW7	0.485	0.745	0.846	0.896	0.919	0.914	1	0.911	0.955	0.172	0.915
SW8	0.542	0.807	0.893	0.913	0.923	0.941	0.911	1	0.947	0.213	0.931
SW9	0.486	0.769	0.868	0.904	0.94	0.925	0.955	0.947	1	0.174	0.927
SWp	0.735	0.562	0.438	0.359	0.323	0.278	0.172	0.213	0.174	1	0.442
R	0.754	0.934	0.972	0.965	0.969	0.954	0.915	0.931	0.927	0.442	1

**Table 2:** The similarity result of Shu Dihuang processed with yellow rice wine determined at 284 nm.

	SW1	SW2	SW3	SW4	SW5	SW6	SW7	SW8	SW9	SWp	R
SW1	1	0.888	0.742	0.242	0.514	0.477	0.306	0.308	0.376	0.679	0.734
SW2	0.888	1	0.918	0.281	0.74	0.713	0.577	0.564	0.63	0.654	0.894
SW3	0.742	0.918	1	0.352	0.85	0.857	0.777	0.714	0.769	0.548	0.949
SW4	0.242	0.281	0.352	1	0.459	0.407	0.38	0.406	0.342	0.121	0.472
SW5	0.514	0.74	0.85	0.459	1	0.917	0.847	0.838	0.817	0.402	0.907
SW6	0.477	0.713	0.857	0.407	0.917	1	0.902	0.881	0.887	0.373	0.911
SW7	0.306	0.577	0.777	0.38	0.847	0.902	1	0.831	0.915	0.26	0.835
SW8	0.308	0.564	0.714	0.406	0.838	0.881	0.831	1	0.806	0.263	0.795
SW9	0.376	0.63	0.769	0.342	0.817	0.887	0.915	0.806	1	0.324	0.853
SWp	0.679	0.654	0.548	0.121	0.402	0.373	0.26	0.263	0.324	1	0.61
R	0.734	0.894	0.949	0.472	0.907	0.911	0.835	0.795	0.853	0.61	1

**Table 3:** The similarity result of Shu Dihuang determined at 205 nm.

	S1	S2	S3	S4	S5	S6	S7	S8	S9	Sp	R
S1	1	0.671	0.798	0.852	0.692	0.655	0.677	0.672	0.606	0.954	0.816
S2	0.671	1	0.896	0.879	0.966	0.95	0.933	0.932	0.941	0.759	0.95
S3	0.798	0.896	1	0.892	0.919	0.911	0.889	0.906	0.894	0.804	0.952
S4	0.852	0.879	0.892	1	0.916	0.868	0.893	0.864	0.831	0.897	0.951
S5	0.692	0.966	0.919	0.916	1	0.966	0.973	0.96	0.957	0.754	0.972
S6	0.655	0.95	0.911	0.868	0.966	1	0.972	0.97	0.988	0.717	0.963
S7	0.677	0.933	0.889	0.893	0.973	0.972	1	0.975	0.965	0.719	0.965
S8	0.672	0.932	0.906	0.864	0.96	0.97	0.975	1	0.969	0.718	0.962
S9	0.606	0.941	0.894	0.831	0.957	0.988	0.965	0.969	1	0.679	0.945
Sp	0.954	0.759	0.804	0.897	0.754	0.717	0.719	0.718	0.679	1	0.858
R	0.816	0.95	0.952	0.951	0.972	0.963	0.965	0.962	0.945	0.858	1

**Table 4:** The similarity result of Shu Dihuang determined at 284 nm.

	S1	S2	S3	S4	S5	S6	S7	S8	S9	Sp	R
S1	1	0.57	0.507	0.595	0.423	0.394	0.384	0.299	0.326	0.823	0.781
S2	0.57	1	0.73	0.656	0.882	0.865	0.846	0.23	0.244	0.44	0.824
S3	0.507	0.73	1	0.873	0.778	0.803	0.78	0.344	0.38	0.404	0.854
S4	0.595	0.656	0.873	1	0.66	0.655	0.65	0.293	0.31	0.506	0.828
S5	0.423	0.882	0.778	0.66	1	0.948	0.92	0.269	0.28	0.24	0.796
S6	0.394	0.865	0.803	0.655	0.948	1	0.93	0.304	0.327	0.262	0.808
S7	0.384	0.846	0.78	0.65	0.92	0.93	1	0.341	0.328	0.248	0.802
S8	0.299	0.23	0.344	0.293	0.269	0.304	0.341	1	0.3	0.472	0.542
S9	0.326	0.244	0.38	0.31	0.28	0.327	0.328	0.3	1	0.215	0.497
Sp	0.823	0.44	0.404	0.506	0.24	0.262	0.248	0.472	0.215	1	0.696
R	0.781	0.824	0.854	0.828	0.796	0.808	0.802	0.542	0.497	0.696	1

**Table 5:** Parameters of pyrolysis characteristics of Shu Dihuang.

Sample	Thermal Decomposition Periods					Mass/(%)		DTG_max_/(%·min^−1^)		
SW1	Water loss (Room temperature~165 °C)					4.03		1.21		
	Pyrolysis & Combustion			165~270 °C		19.20		8.33		
				270~390 °C		24.22		2.75		
	Carbonization & Combustion (390 °C~Final temperature)					21.50		8.83		
SW2	Water loss (Room temperature~165 °C)					4.27		1.34		
	Pyrolysis & Combustion			165~265 °C		18.94		7.68		
				265~395 °C		24.23		2.78		
	Carbonization & Combustion (395 °C~Final temperature)					21.97		10.07		
SW3	Water loss (Room temperature~155 °C)					4.34		1.34		
	Pyrolysis & Combustion			155~270 °C		18.27		6.47		
				270~390 °C		22.15		2.46		
	Carbonization & Combustion (390 °C~Final temperature)					21.05		5.07		
SW4	Water loss (Room temperature~155 °C)					3.80		1.31		
	Pyrolysis & Combustion			155~270 °C		17.32		6.11		
				270~390 °C		24.62		2.58		
	Carbonization & Combustion (390 °C~Final temperature)					23.27		4.47		
SW5	Water loss (Room temperature~145 °C)					3.20		1.20		
	Pyrolysis & Combustion			145~265 °C		16.56		5.84		
				265~385 °C		24.78		2.77		
	Carbonization & Combustion (385 °C~Final temperature)					26.55		5.51		
SW6	Water loss (Room temperature~150 °C)					3.65		1.15		
	Pyrolysis & Combustion			150~270 °C		15.62		5.21		
				270~385 °C		24.22		2.81		
	Carbonization & Combustion (385 °C~Final temperature)					25.28		5.46		
SW7	Water loss (Room temperature~150 °C)					3.73		1.23		
	Pyrolysis & Combustion			150~270 °C		15.38		5.30		
				270~385 °C		24.56		2.67		
	Carbonization & Combustion (385 °C~Final temperature)					25.70		5.03		
SW8	Water loss (Room temperature~145 °C)					3.99		1.18		
	Pyrolysis & Combustion			145~270 °C		14.51		4.88		
				270~380 °C		22.93		2.96		
	Carbonization & Combustion (380 °C~Final temperature)					23.97		4.68		
SW9	Water loss (Room temperature~145 °C)					3.61		1.19		
	Pyrolysis & Combustion			145~270 °C		14.62		4.85		
				270~385 °C		23.85		2.90		
	Carbonization & Combustion (385 °C~Final temperature)					25.44		5.45		
S1	Water loss (Room temperature~170 °C)					4.42		0.91		
	Pyrolysis & Combustion			170~260 °C		16.29		10.57		
				260~385 °C		25.53		2.93		
	Carbonization & Combustion (385 °C~Final temperature)					19.84		5.71		
S2	Water loss (Room temperature~160 °C)					1.92		0.62		
	Pyrolysis & Combustion			160~270 °C		15.82		8.20		
				270~380 °C		26.36		3.00		
	Carbonization & Combustion (380 °C~Final temperature)					19.92		5.15		
S3	Water loss (Room temperature~155 °C)					2.94		0.96		
	Pyrolysis & Combustion			155~270 °C		17.58		7.23		
				270~385 °C		24.35		2.70		
	Carbonization & Combustion (385 °C~Final temperature)					21.18		4.47		
S4	Water loss (Room temperature~155 °C)					4.99		0.82		
	Pyrolysis & Combustion			155~270 °C		13.91		6.63		
				270~380 °C		23.49		2.72		
	Carbonization & Combustion (380 °C~Final temperature)					21.69		4.35		
S5	Water loss (Room temperature~150 °C)					6.28		1.28		
	Pyrolysis & Combustion			150~270 °C		12.85		7.55		
				270~380 °C		24.70		2.66		
	Carbonization & Combustion (380 °C~Final temperature)					20.70		4.10		
S6	Water loss (Room temperature~160 °C)					5.42		0.91		
	Pyrolysis & Combustion			160~270 °C		13.37		7.05		
				270~380 °C		24.25		2.68		
	Carbonization & Combustion (380 °C~Final temperature)					31.20		3.09		
S7	Water loss (Room temperature~160 °C)					5.02		1.00		
	Pyrolysis & Combustion			160~270 °C		13.56		6.29		
				270~370 °C		23.65		2.78		
	Carbonization & Combustion (370 °C~Final temperature)					21.52		2.98		
S8	Water loss (Room temperature~160 °C)					5.10		0.98		
	Pyrolysis & Combustion			160~275 °C		13.87		5.86		
				275~375 °C		23.61		2.68		
	Carbonization & Combustion (375 °C~Final temperature)					21.97		3.27		
S9	Water loss (Room temperature~170 °C)					6.55		1.17		
	Pyrolysis & Combustion			170~255 °C		12.17		5.84		
				255~380 °C		24.20		3.03		
	Carbonization & Combustion (380 °C~Final temperature)					21.96		5.10		
SWP	Water loss (Room temperature~170 °C)					4.00		1.23		
	Pyrolysis & Combustion			170~270 °C		20.05		9.65		
				270~370 °C		24.18		2.78		
	Carbonization & Combustion (370 °C~Final temperature)					23.08		6.27		
SP	Water loss (Room temperature~175 °C)					7.17		2.04		
	Pyrolysis & Combustion			175~260 °C		18.12		9.19		
				260~390 °C		24.39		2.90		
	Carbonization & Combustion (390 °C~Final temperature)					20.99		9.83		

**Table 6:** The FTIR similarity result of Shu Dihuang.

	S1	S2	S3	S4	S5	S6	S7	S8	S9	SP
S1	1	0.835	0.696	0.428	0.676	0.626	0.691	0.534	0.640	0.871
S2	0.835	1	0.842	0.666	0.926	0.729	0.859	0.809	0.838	0.716
S3	0.696	0.842	1	0.912	0.805	0.919	0.956	0.898	0.860	0.539
S4	0.428	0.666	0.912	1	0.709	0.915	0.901	0.887	0.784	0.285
S5	0.676	0.926	0.805	0.709	1	0.706	0.843	0.873	0.841	0.520
S6	0.626	0.729	0.919	0.915	0.706	1	0.945	0.804	0.789	0.549
S7	0.691	0.859	0.956	0.901	0.843	0.945	1	0.878	0.856	0.608
S8	0.534	0.809	0.898	0.887	0.873	0.804	0.878	1	0.832	0.331
S9	0.640	0.838	0.860	0.784	0.841	0.789	0.856	0.832	1	0.489
SP	0.871	0.716	0.539	0.285	0.520	0.549	0.608	0.331	0.489	1

**Table 7:** The FTIR similarity result of Shu Dihuang processed with yellow rice wine.

	SW1	SW2	SW3	SW4	SW5	SW6	SW7	SW8	SW9	SWP
SW1	1	0.777	0.808	0.751	0.918	0.932	0.921	0.750	0.744	0.864
SW2	0.777	1	0.730	0.676	0.800	0.814	0.757	0.673	0.507	0.674
SW3	0.808	0.730	1	0.449	0.792	0.828	0.910	0.418	0.545	0.844
SW4	0.751	0.676	0.449	1	0.860	0.812	0.717	0.882	0.802	0.723
SW5	0.918	0.800	0.792	0.860	1	0.955	0.947	0.788	0.859	0.925
SW6	0.932	0.814	0.828	0.812	0.955	1	0.947	0.809	0.770	0.896
SW7	0.921	0.757	0.910	0.717	0.947	0.947	1	0.662	0.791	0.951
SW8	0.750	0.673	0.418	0.882	0.788	0.809	0.662	1	0.664	0.623
SW9	0.744	0.507	0.545	0.802	0.859	0.770	0.791	0.664	1	0.837
SWP	0.864	0.674	0.844	0.723	0.925	0.896	0.951	0.623	0.837	1

**Table 8:** The HPLC mobile phase gradient program.

T/min	0.1% Phosphoric Acid/%	Acetonitrile/%
0	100	0
5	100	0
10	98	2
22	96	4
40	85	15
50	80	20
60	75	25

## Supplementary Materials

Supplementary File 1
