# Supplementary material for: Investigation of the Effect of the Degree of Processing of Radix Rehmanniae Preparata (Shu Dihuang) on Shu Dihuangtan Carbonization Preparation Technology
Source: Molecules. 2017 Jul 18;22(7):1193. doi: 10.3390/molecules22071193 (PMC6152270; doi:10.3390/molecules22071193)
Supplement: Supplementary File 1 [file molecules-22-01193-s001.pdf]

熟地黄炮制（九蒸九晒）过程中药效化学成分量变化及炮制辅料对其影响研究

孟祥龙，马俊楠，张朔生*，李 坤，薛非非，张 洁，原志翠，田叶飞，王明芳
山西中医学院制药与食品工程学院，山西 晋中 030619

摘 要：目的 基于 HPLC 法探讨炮制辅料黄酒对九蒸九晒制备得到的熟地黄的化学成分动态变化的影响差异性。**方法** 色谱柱为 Hypersil GOLD aQ C_{18} 色谱柱（250 mm×4.6 mm，5 μm），体积流量为 1 mL/min，柱温为 25 ℃。梓醇定量测定：流动相为乙腈-0.1%磷酸水（1∶99），检测波长为 210 nm；地黄苷 A 定量测定：流动相为乙腈-0.1%磷酸水（3∶97），检测波长为 203 nm；地黄苷 D 和益母草苷定量测定：流动相为乙腈-0.1%磷酸水（4∶96），检测波长为 203 nm；毛蕊花糖苷定量测定：流动相为乙腈-0.1%磷酸水（16∶84），检测波长为 334 nm；5-羟甲基糠醛（5-HMF）定量测定：流动相为乙腈-0.1%磷酸水（11∶89），检测波长为 284 nm。糖类成分的测定：色谱柱为 Accurasil NH_2 柱（250 mm×4.6 mm，5 μm），流动相为乙腈-水（72∶28），体积流量为 1 mL/min，柱温 25 ℃，检测器为 ELSD，Alltech2000ES，漂移管温度 93 ℃，气体体积流量 2.6 L/min。采用 SPSS 20.0 进行统计分析，将清蒸-九蒸九晒与酒蒸-九蒸九晒制得的不同蒸晒次数的熟地黄中 12 种化学成分定量测定数据进行配对样本 *t*-检验。**结果** 随蒸晒次数的增加，清蒸法与酒蒸法制得熟地黄中梓醇及益母草苷、毛蕊花糖苷、水苏糖、蔗糖、棉子糖均减少；地黄苷 A 及地黄苷 D 略为增加；5-HMF、果糖、葡萄糖、甘露三糖均增加。**结论** 建立的方法符合方法学验证要求。在古法熟地黄九蒸九晒的炮制过程中，炮制辅料黄酒对炮制品的质量存在显著影响，且清蒸法与酒蒸法均以第 3 次、第 4 次及第 6 次蒸晒所得熟地黄的相关物质的量呈较大的波动性。

关键词：熟地黄；中药炮制；九蒸九晒；炮制辅料；梓醇；地黄苷 A；地黄苷 D；益母草苷；毛蕊花糖苷；5-羟甲基糠醛；糖类成分
中图分类号：R283.1 **文献标志码：**A **文章编号：**0253 - 2670(2016)05 - 0752 - 08
DOI: 10.7501/j.issn.0253-2670.2016.05.011

Content changes of chemical components and their effect of adjuvants during the process of *Rehmanniae Radix Praeparata* (steamed for nine times and shined for nine times)

MENG Xiang-long, MA Jun-nan, ZHANG Shuo-sheng, LI Kun, XUE Fei-fei, ZHANG Jie, YUAN Zhi-cui, TIAN Ye-fei, WANG Ming-fang
Institute of Pharmaceutical & Food Engineering, Shanxi University of Traditional Chinese Medicine, Jinzhong 030619, China

Abstract: Objective By HPLC, to explore the differences of adjuvants for the changes of chemical components of *Rehmanniae Radix Praeparata* during process by steamed for nine times and shined for nine times. **Methods** HPLC was conducted on a Hypersil GOLD aQ C_{18} column (250 mm × 4.6 mm, 5 μm), flow rate was 1 mL/min, and the column temperature was 25 ℃. With the mobile phase of acetonitrile and 0.1% phosphoric acid water (1∶99), the detection wavelength was 210 nm (catalpol), 203 nm (rehmannioside A, rehmannioside D, and leonuride), acetonitrile and 0.1% phosphoric acid water (3∶97 and 4∶96); 334 nm (actecosode), acetonitrile and 0.1% phosphoric acid water (16∶84), and 284 nm (5-HMF), acetonitrile and 0.1% phosphoric acid water (11∶89). Sugars was conducted on an Accurasil NH_2 column (250 mm × 4.6 mm, 5 μm) with the mobile phase of acetonitrile and 0.1% phosphoric acid water (72∶28), flow rate was 1 mL/min, the column temperature was 25 ℃. The detector was ELSD, Alltech2000ES, drift tube temperature was 93 ℃, and the gas flow rate was 2.6 L/min. The statistic analysis was carried out by SPSS 20.0 and the paired sample *t*-test was carried out for 12 kinds of chemical composition in *Rehmanniae Radix Praeparata* obtained by steamed for nine times and shined for nine times and wine-steamed for nine times and shined for nine times. **Results** With the increase of steamed times, the

收稿日期：2015-10-01
基金项目：山西省中药现代化关键技术研究振东专项（2014ZD0302）
作者简介：孟祥龙，男，助教，主要从事药食两用中药质量标准及炮制现代研究。E-mail: sszywzh@126.com
*通信作者 张朔生，男，教授，硕士生导师，主要从事中药饮片炮制工艺及质量标准研究。E-mail: zhangshuosheng@aliyun.com

contents of catalpol, leonuride, actecosode, stachyose, sucrose, and raffinose in *Rehmanniae Radix Praeparata* obtained by two ways are all decreased; While the contents of rehmannioside A and rehmannioside D were slightly increased; the contents of 5-HMF, fructose, glucose, and mannose trisaccharide were increased. **Conclusion** The established method conforms to the validation requirement of the methodology. In the process of *Rehmanniae Radix Praeparata* with steamed for nine times and shined for nine times, processing adjuvant has a marked impact on the quality of the processed product. And the related substances of *Rehmanniae Radix Praeparata* obtained by the two ways have a larger fluctuation in times 3, 4, and 6 of steamed and shined processes, and the reason for this still needs to be further studied.

Key words: *Rehmanniae Radix Praeparata*; Chinese materia medica process; steamed for nine times and shined for nine times; adjuvants; catalpolreh; mannioside A; rehmannioside D; leonuride; actecosode; 5-HMF; sugars

地黄 *Rehmanniae Radix* 为我国“四大怀药”之一，始载于《神农本草经》[1]，为玄参科（Scrophulariaceae）地黄属 *Rehmannia* Libosch. ex Fisch. et Mey. 植物地黄 *Rehmannia glutinosa* Libosch. 干燥块根。临床常用的规格为鲜地黄、生地黄、熟地黄、地黄炭及熟地黄炭等，《中国药典》2010 年版地黄及熟地黄[2]项下共收载鲜地黄、生地黄、酒炖地黄、清蒸地黄 4 种炮制品，其生熟之品性味功效迥异。

熟地黄的炮制加工方法始载于汉代《金匮要略方论》[3]，后世历代本草对熟地黄的加工炮制工艺记载及争议颇多，主要为加工方法和所用辅料的不同、蒸制次数及时间等方面[4]。熟地黄制法各异，然皆以“光黑如漆，味甘如饴”为质量评价、以“九蒸九晒”为达到炮制时间较长的制作要求，同时在传统经验上还符合“太过则性味发酸”的准则。

针对九蒸九晒制备熟地黄，虽卢鹏伟[5]对九蒸九晒法炮制过程中环烯醚萜类成分的量变化进行研究，郭艳霞[6]尝试对长时间炮制制备熟地黄中美拉德反应的程度研究，但相关典籍及现代研究皆缺少炮制辅料黄酒对九蒸九晒制备熟地黄化学成分动态变化的影响差异研究。故本实验以此为突破口，以期阐释结合炮制辅料的九蒸九晒法所制备熟地黄的炮制科学内涵。

1 仪器与材料

Thermo Fisher Scientific U3000 高效液相色谱仪、GZX-9076MBE 电热鼓风干燥箱、HHS 恒温水浴锅，上海博讯实业有限公司医疗设备厂；DV215CD 万分之一精密电子天平、超声波清洗机，宁波新芝生物科技股份有限公司。

生地黄：采自山西省临汾市襄汾县，经山西中医学院制药与食品工程学院张朔生教授鉴定为玄参科地黄属植物地黄 *Rehmannia glutinosa* Libosch. 的块根。

清蒸-九蒸九晒熟地黄[7]：取生地黄，蒸锅蒸制 6 h，取出，置干燥箱内，60 ℃干燥 24 h，为一蒸一晒熟地黄，反复蒸制 9 次，分别得到不同蒸晒次数的熟地黄样品。切制，60 ℃干燥即得，分别记为 S_1～S_9。

酒蒸-九蒸九晒熟地黄[5,8]：取生地黄，加黄酒拌制（药材与黄酒的比例为 10∶4），待黄酒吸干后，按清蒸-九蒸九晒熟地黄制法操作得样品，分别记为 SW_1～SW_9。

塔牌花雕酒（浙江塔牌绍兴酒有限公司），乙腈为色谱纯，水为纯化水，甲醇、磷酸为分析纯。

对照品地黄苷 A（批号 130506，质量分数≥98%）、地黄苷 D（批号 140402，质量分数≥98%）、益母草苷（批号 130911，质量分数≥98%）、梓醇（批号 150331，质量分数≥98%）、5-羟甲基糠醛（5-HMF，批号 14099，质量分数≥98%）、半乳糖（批号 100226-201105，质量分数≥98%）、无水葡萄糖（批号 111507-201303，质量分数≥98%）、甘露三糖（批号 140328，质量分数≥98%），成都普菲德生物科技有限公司；对照品毛蕊花糖苷（批号 MST-13122711，质量分数≥98%）、果糖（批号 MST-13072803，质量分数≥98%）、蔗糖（批号 MST-13122601，质量分数≥98%），成都曼斯特生物科技有限公司；棉子糖（批号 130513，质量分数≥98%），深圳振强生物技术有限公司；水苏糖（批号 14070310，质量分数≥98%），上海拜力生物有限公司。

2 方法与结果

2.1 环烯醚萜类成分定量测定

2.1.1 供试品溶液的制备 取样品 S_1～S_9 及 SW_1～SW_9 各 0.5 g 置于 50 mL 量瓶中，加甲醇定容，置于室温下冷浸过夜后超声提取 1.5 h，冷却至室温，补足减失的质量，滤过，取续滤液 25 mL，蒸干，残渣用 15%甲醇溶解，定容至 5 mL。用 0.45 μm 微

孔滤膜滤过，即得供试品溶液。所有样品平行制备 3 份。

2.1.2　对照品溶液的制备　精密称取干燥至恒定质量的梓醇、益母草苷、地黄苷 D、地黄苷 A 对照品适量，加甲醇定容至 5 mL。分别制成含梓醇 202.4 μg/mL、益母草苷 543 μg/mL、地黄苷 D 78.2 μg/mL、地黄苷 A 83 μg/mL 的混合对照品溶液。

另取甲醇定容至 5 mL 作为空白对照。

2.1.3　色谱条件　色谱柱为 Hypersil GOLD aQ C_{18} 色谱柱（250 mm×4.6 mm，5 μm），体积流量为 1 mL/min，柱温为 25 ℃。熟地黄梓醇定量测定：流动相为乙腈-0.1%磷酸水（1∶99），检测波长为 210 nm；地黄苷 A 定量测定：流动相为乙腈-0.1%磷酸水（3∶97），检测波长为 203 nm；地黄苷 D 和益母草苷定量测定：流动相为乙腈-0.1%磷酸水（4∶96），检测波长为 203 nm。色谱图见图 1。

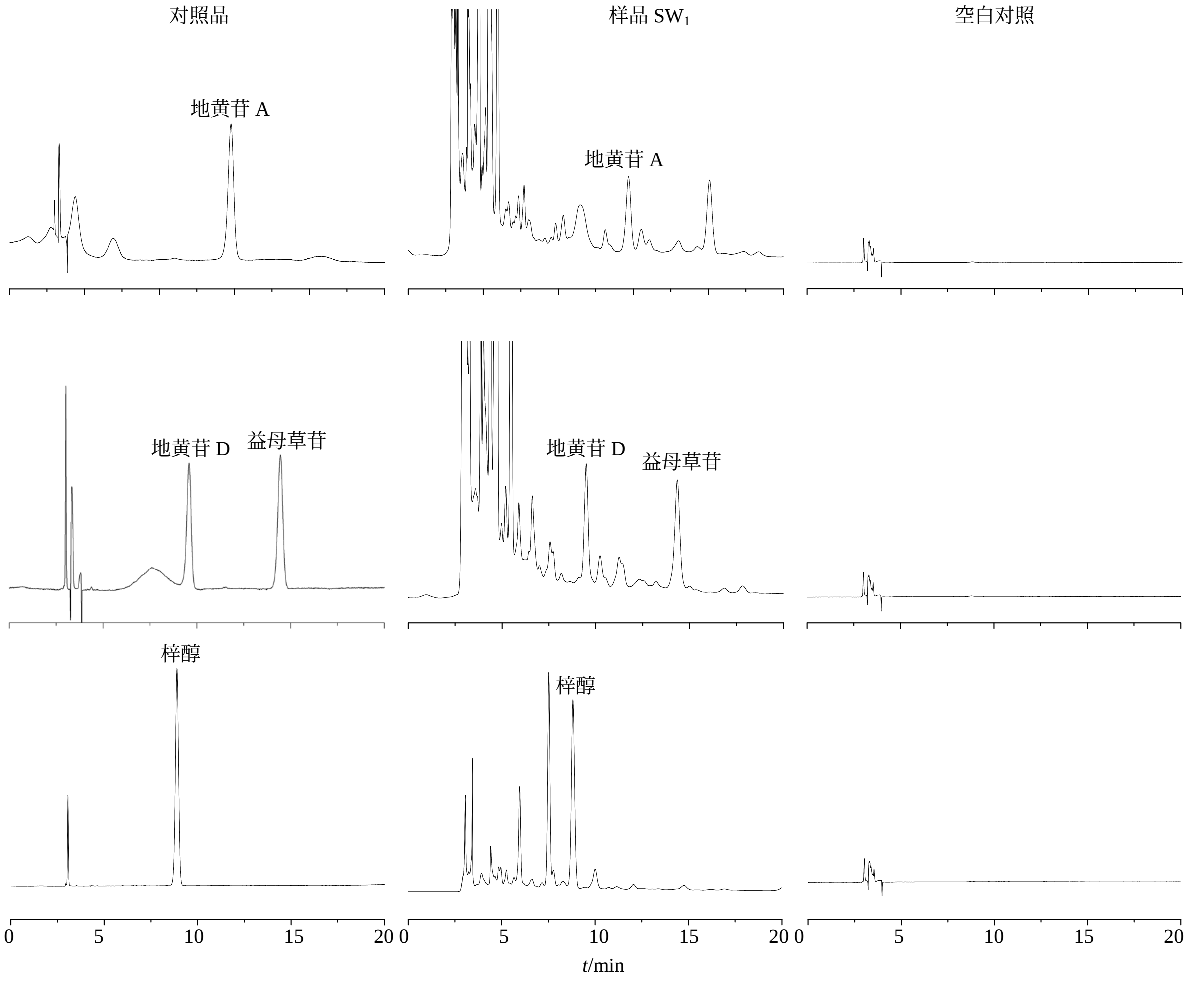

图 1　环烯醚萜类对照品与样品 SW_1 的 HPLC 图谱

Fig. 1　HPLC for reference substance of iridoid and sample SW_1

2.1.4　线性关系考察　将“2.1.2”项下各对照品分别配成梓醇质量浓度为 2 024、404.8、202.4、80.96、40.48 μg/mL 的溶液；益母草苷质量浓度为 5 430、1 086、543、217.2、108.6 μg/mL 的溶液；地黄苷 A 质量浓度为 830、166、83、33.2、16.6 μg/mL 的溶液；地黄苷 D 质量浓度为 782、156.4、78.2、31.28、15.64 μg/mL 的溶液。按上述色谱条件各进样 10 μL，测定峰面积。将所得峰面积积分值与各对照品的质量浓度进行线性回归，得回归方程、相关系数（r）及线性范围分别为梓醇：$Y=106.7\ X-14.58$，$r=0.999\ 4$，2 024～40.48 μg/mL；益母草苷：$Y=115.4\ X-0.641$，$r=0.999\ 5$，5 430～108.6 μg/mL；地黄苷 A：$Y=30.20\ X-2.065$，$r=0.999\ 5$，830～16.6 μg/mL；地黄苷 D：$Y=53.52\ X-10.02$，$r=0.999\ 4$，782～15.64 μg/mL。

2.1.5　方法学考察　依照方法学考察要求，分别进行精密度、稳定性、重复性及加样回收率考察，结果分别为精密度：梓醇峰面积 RSD 为 0.74%，益母

草苷峰面积 RSD 为 0.60%，地黄苷 A 峰面积 RSD 为 0.51%，地黄苷 D 峰面积 RSD 为 1.55%；分别于 1、2、4、8、16、24 h 测定同一份供试品溶液（SW_1）的稳定性，得梓醇峰面积 RSD 为 1.55%，益母草苷峰面积 RSD 为 2.07%，地黄苷 A 峰面积 RSD 为 2.08%，地黄苷 D 峰面积 RSD 为 2.04%；取同批供试品溶液（SW_1），依法平行测定 6 份，考察重复性，梓醇质量分数 RSD 为 1.69%，益母草苷质量分数 RSD 为 1.06%，地黄苷 A 质量分数 RSD 值 0.30%，地黄苷 D 质量分数 RSD 为 0.16%；鉴于对照品量有限，仅对梓醇做加样回收率的考察，得梓醇平均回收率为 100.59%，RSD 值为 1.59%。

2.1.6 样品定量测定 取“2.1.1”项下制备的 S_1～S_9 及 SW_1～SW_9 供试品溶液，依法进行测定，结果见表 1。

2.2 毛蕊花糖苷定量测定

2.2.1 供试品溶液的制备 制法同“2.1.1”项下。

2.2.2 对照品溶液的制备 精密称取干燥至恒定质量的毛蕊花糖苷对照品适量，加纯水定容至 10 mL，制成含毛蕊花糖苷 200 μg/mL 的对照品溶液。

另取纯水定容至 10 mL 作为空白对照。

2.2.3 色谱条件 色谱柱为 Hypersil GOLD aQC_{18} 柱（250 mm×4.6 mm, 5 μm），体积流量为 1 mL/min，柱温为 25 ℃；流动相为乙腈-0.1%磷酸水溶液（16∶84），检测波长为 334 nm。色谱图见图 2。

2.2.4 线性关系考察 将毛蕊花糖苷对照品溶液分别配成质量浓度为 200、80、40、8、1.6 μg/mL 的溶液。按上述色谱条件各进样 10 μL，测定峰面积，将所得峰面积与毛蕊花糖苷质量浓度进行线性回归，得回归方程 $Y=5.865X+0.655$，$r=0.999\ 5$。

2.2.5 方法学考察 依照方法学考察要求，分别进行精密度、稳定性、重复性及加样回收率考察，结果分别为精密性：得毛蕊花糖苷峰面积 RSD 为 0.84%；分别于 1、2、4、8、16、24 h 测定同一份

表 1 熟地黄中环烯醚萜类成分随蒸晒次数的动态变化情况 (*n* = 3)

Table 1 Changes of iridoid contents affected by steamed times in process of *Rehmanniae Radix Praeparata* (*n* = 3)

蒸晒次数	梓醇/(μg·g^{-1})		地黄苷 A/(μg·g^{-1})		地黄苷 D/(μg·g^{-1})		益母草苷/(μg·g^{-1})	
	S	SW	S	SW	S	SW	S	SW
1	51 350.45	58 905.24	961.19	1 252.98	646.20	564.70	14 104.99	17 399.19
2	21 745.04	34 854.42	985.65	1 212.66	625.99	589.41	5 597.35	12 465.96
3	12 429.39	18 419.10	801.82	1 526.44	692.12	620.04	4 997.16	5 982.09
4	9 176.74	5 913.00	999.72	1 264.70	712.94	633.00	3 640.63	3 152.14
5	4 215.94	3 589.29	1 017.12	1 355.93	823.02	801.11	2 340.53	—
6	3 485.69	2 386.46	953.94	1 446.83	873.54	830.62	2 142.97	—
7	2 645.10	3 191.62	1 035.30	1 884.04	971.35	1 142.00	1 962.71	—
8	1 428.94	2 456.99	1 043.18	1 797.94	1 130.11	1 448.08	—	—
9	1 458.28	2 751.91	954.06	634.70	878.23	1 136.67	—	—

S 代表清蒸品；SW 代表酒蒸品，下同

S stands for steamed product; SW stands for wine-steamed product, same as below

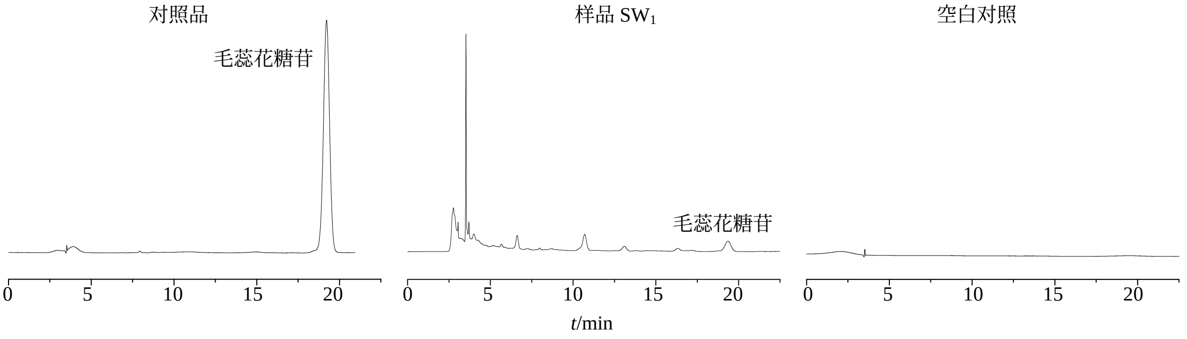

图 2 毛蕊花糖苷对照品与样品 SW_1 的 HPLC 图谱

Fig. 2 HPLC for reference substance of acteoside and sample SW_1

供试品溶液（SW_1）的稳定性，得毛蕊花糖苷峰面积 RSD 为 1.03%；取同批供试品溶液（SW_1），依法平行测定 6 份，考察重复性，得到毛蕊花糖苷质量分数 RSD 为 1.47%；加样回收率，得毛蕊花糖苷平均回收率为 98.91%，RSD 值为 0.86%。

2.2.6 样品定量测定 取“2.1.1”项下制备的 S_1～S_9 及 SW_1～SW_9 供试品溶液，依法进行测定，结果见表 2。

表 2 毛蕊花糖苷和 5-HMF 随蒸晒次数的动态变化情况

Table 2 Changes of acteoside and 5-HMF contents affected by steamed times in process of *Rehmanniae Radix Praeparata*

蒸晒次数	毛蕊花糖苷/(μg·g^{-1})		5-HMF/(μg·g^{-1})	
	S	SW	S	SW
1	139.07	214.86	82.15	40.56
2	139.61	215.82	168.89	62.75
3	85.10	94.04	195.77	102.99
4	57.59	92.15	275.09	238.63
5	43.15	93.39	453.99	423.34
6	30.38	160.48	593.81	524.18
7	34.33	67.08	761.41	1 077.66
8	—	59.15	952.13	1 493.93
9	—	56.72	752.63	1 326.68

2.3 5-HMF 定量测定

2.3.1 供试品溶液的制备 制法同“2.1.1”项下。

2.3.2 对照品溶液的制备 精密称取干燥至恒定质量的 5-HMF 对照品适量，加甲醇定容至 10 mL。制成含 5-HMF 2 276 μg/mL 的对照品溶液。

另取甲醇定容至 5 mL 作为空白对照。

2.3.3 色谱条件 色谱柱为 Hypersil GOLD aQ C_{18} 色谱柱（250 mm×4.6 mm，5 μm），体积流量为 1 mL/min，柱温为 25 ℃。流动相为乙腈-0.1%磷酸水溶液（11∶89），检测波长为 284 nm。色谱图见图 3。

2.3.4 线性关系考察 取“2.3.2”项下对照品溶液分别配成质量浓度为 227.6、113.8、45.52、9.104、0.455 2 μg/mL 的溶液，各进样 10 μL，测定峰面积，将峰面积与 5-HMF 质量浓度进行线性回归，得回归方程 $Y=1.078X+0.471$，$r=0.999\,4$。

2.3.5 方法学考察 依照方法学考察要求，分别进行精密度、稳定性、重复性及加样回收率考察，结果分别为精密度：得 5-HMF 峰面积 RSD 为 1.34%；分别于 1、2、4、8、16、24 h 测定同一份供试品溶液（SW_1）的稳定性，得 5-HMF 峰面积 RSD 为 2.02%；取同批供试品溶液（SW_1），依法平行测定 6 份，考察重复性，得 5-HMF 质量分数 RSD 为 0.61%；另鉴于对照品量有限，所以本项下未进行加样回收率的测定。

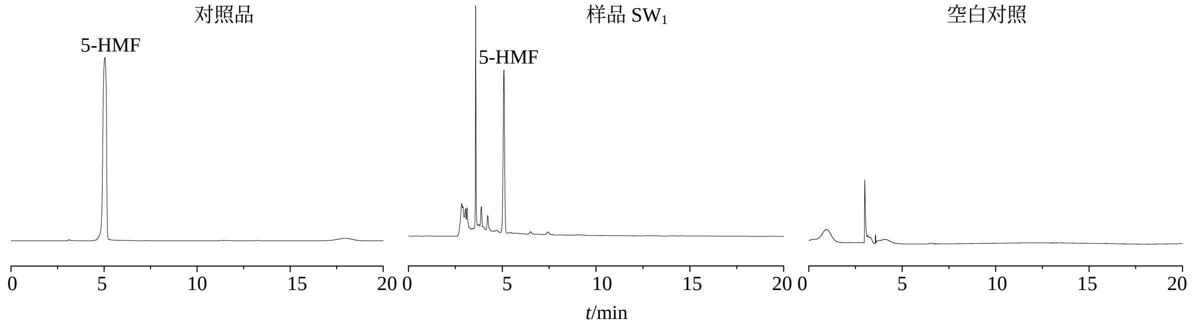

图 3 5-HMF 对照品与样品 SW_1 的 HPLC 图谱

Fig. 3 HPLC for reference substance of 5-HMF and sample SW_1

2.3.6 样品定量测定 取“2.1.1”项下制备的 S_1～S_9 及 SW_1～SW_9 供试品溶液，依法进行定量测定，结果见表 2。

2.4 糖类成分定量测定

2.4.1 供试品溶液的制备 取材料项下 S_1～S_9 及 SW_1～SW_9 样品各 1 g，置锥形瓶中，加入 20 mL 蒸馏水，超声提取 2 h，冷却至室温，补足减失的质量，置于离心管中离心，倾出上清液，置于 PE 管中待用。量取待用液 1 mL 定容至 10 mL。用 0.45 μm 微孔滤膜滤过，即得供试品溶液。

2.4.2 混合对照品溶液制备 精密称取干燥至恒定质量的果糖、无水葡萄糖、半乳糖、蔗糖、棉子糖、甘露三糖、水苏糖对照品适量，加 1%乙腈水定容至 5 mL。制成含果糖 1.162 mg/mL、无水葡萄糖 0.980 mg/mL、半乳糖 1.186 mg/mL、蔗糖 0.970 mg/mL、棉子糖 1.086 mg/mL、甘露三糖 1.560 mg/mL、水苏糖 0.910 mg/mL 的混合对照品溶液。

另取 1%乙腈水定容至 5 mL 做为空白对照。

2.4.3 色谱条件 色谱柱为 Accurasil NH_2 柱（250 mm×4.6 mm，5 μm），流动相为乙腈-水（72∶28），体积流量 1 mL/min，柱温 25 ℃，检测器为 ELSD，Alltech2000ES，漂移管温度 93 ℃，气体体积流量 2.6 L/min。色谱图见图 4。

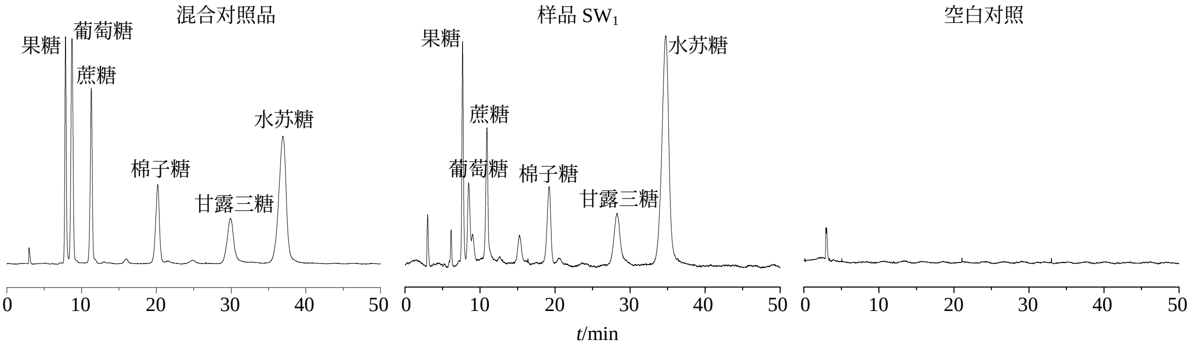

图 4 混合对照品与样品 SW_1 的 HPLC 图谱

Fig. 4 HPLC for mixed reference substance and sample SW_1

2.4.4 线性关系考察 取“2.4.2”项下对照品溶液，分别配成果糖质量浓度为 1 162、232.4、116.2、46.48、23.24 μg/mL 的溶液；葡萄糖质量浓度为 980、196、98、39.2、19.6 μg/mL 的溶液；蔗糖质量浓度为 970、194、97、38.8、19.4 μg/mL 的溶液；棉子糖质量浓度为 1 086、217.2、108.6、43.44、21.72 μg/mL 的溶液；甘露三糖质量浓度为 1 560、312、156、62.4、31.2 μg/mL 的溶液；水苏糖质量浓度为 3 302、910、182、91、36.4、18.2 μg/mL 的溶液。各进样 10 μL，测定峰面积，将峰面积的对数与质量浓度的对数进行线性回归，得回归方程分别为果糖 $Y=0.991\ X+0.355$，$r=0.999\ 4$；葡萄糖 $Y=1.005\ X+0.294$，$r=0.999\ 5$；蔗糖 $Y=0.969\ X+0.329$，$r=0.999\ 5$；棉子糖 $Y=1.021\ X+0.330$，$r=0.999\ 5$；甘露三糖 $Y=0.964\ X+0.801$，$r=0.999\ 5$；水苏糖 $Y=1.001\ X+0.400$，$r=0.999\ 5$。

2.4.5 方法学考察 依照方法学考察要求，分别进行精密度、稳定性、重复性及加样回收率考察，结果分别为精密度，得葡萄糖 RSD 为 2.39%，甘露三糖 RSD 为 1.93%，果糖 RSD 为 0.80%，蔗糖 RSD 为 0.73%，水苏糖 RSD 为 1.23%，棉子糖 RSD 为 0.96%；分别于 1、2、4、8、16、24 h 测定同一份供试品溶液（SW_1）的稳定性，得葡萄糖 RSD 为 6.58%，甘露三糖 RSD 为 0.65%，果糖 RSD 为 2.35%，蔗糖 RSD 为 1.37%，棉子糖 RSD 为 1.84%，水苏糖 RSD 为 1.47%；取同批供试品溶液（SW_1），依法平行测定 6 份，考察重复性，得葡萄糖 RSD 为 6.58%，甘露三糖 RSD 为 0.65%，果糖 RSD 为 2.35%，蔗糖 RSD 为 1.37%，棉子糖 RSD 为 1.84%，水苏糖 RSD 为 1.47%；鉴于对照品量有限，本项下仅对果糖和水苏糖做加样回收率的考察，得果糖平均回收率为 100.59%，RSD 为 0.82%；水苏糖平均回收率 100.66%，RSD 为 1.39%。

2.4.6 样品定量测定 取“2.4.1”项下制备的 S_1～S_9 及 SW_1～SW_9 供试品溶液，依法进行测定，结果见表 3。

2.5 结果分析

采用 SPSS 20.0 进行统计分析，将清蒸法和酒蒸法制得的不同蒸晒次数的熟地黄中 12 种化学成分定量测定数据进行配对样本 *t* 检验，统计处理结果见表 4～6。

比较清蒸法与酒蒸法对熟地黄九蒸九晒的影响可知，就环烯醚萜类成分而言，单糖苷如梓醇及益母草苷的量均伴随蒸晒次数的增加而减小，三糖苷如地黄苷 A 及地黄苷 D 伴随蒸晒次数的增加略为升高。清蒸法与酒蒸法经不同蒸晒次数所制备的熟地黄整体差异性强弱依次为地黄苷 A、益母草苷、梓醇及地黄苷 D，具体表现为酒蒸法熟地黄的地黄苷 A 的量大于清蒸法，且于第 3 次蒸晒时清蒸法与酒蒸法制得的熟地黄中地黄苷 A 的量存在波动；在第 1 至第 4 次蒸晒间清蒸法所制备的熟地黄地黄苷 D 的量略大于相应酒蒸法，第 5 次蒸晒后，酒蒸法熟地黄的地黄苷 D 的量呈大于清蒸法的趋势；第 1 至第 3 次蒸晒过程中，酒蒸法熟地黄的梓醇及益母草苷的量略大于清蒸法，然在之后的蒸晒中，酒蒸法与清蒸法梓醇的量差异不大，且酒蒸法中益母草苷的量快速降低。

就苯乙醇苷类成分而言，毛蕊花糖苷的量伴随

表 3 熟地黄中糖类成分随蒸晒次数的动态变化情况

Table 3 Changes of suger contents affected by steamed times in process of *Rehmanniae Radix Praeparata*

蒸晒次数	果糖/(μg·g^{-1})		葡萄糖/(μg·g^{-1})		蔗糖/(μg·g^{-1})		棉子糖/(μg·g^{-1})		甘露三糖/(μg·g^{-1})		水苏糖/(μg·g^{-1})	
	S	SW	S	SW	S	SW	S	SW	S	SW	S	SW
1	1 244.34	1 342.86	889.39	1 256.93	3 444.40	2 493.34	2 745.15	3 323.68	2 033.87	3 417.78	20 941.32	18 495.85
2	3 777.67	2 601.72	1 721.46	1 617.71	1 846.55	1 892.06	1 753.45	2 675.08	9 310.77	5 490.91	12 633.88	14 749.05
3	4 896.30	4 604.26	2 494.56	2 190.48	1 237.44	1 202.16	1 246.53	1 579.86	13 028.86	11 001.25	8 214.10	9 490.55
4	4 489.53	6 085.33	2 144.32	2 636.56	1 454.21	532.72	1 329.35	691.39	11 347.29	14 466.37	9 778.30	3 869.41
5	6 386.24	6 352.50	3 026.55	2 821.68	468.04	299.43	442.65	357.04	16 988.69	16 222.93	2 441.02	2 060.96
6	6 232.09	6 216.69	3 175.36	2 831.40	360.01	—	—	—	18 701.57	15 865.12	1 789.98	941.81
7	5 759.09	5 938.34	3 280.79	3 291.41	238.03	—	—	—	19 077.31	15 252.61	1 072.82	—
8	5 560.56	5 269.20	3 281.94	2 870.45	—	—	—	—	18 463.25	13 879.48	—	—
9	5 048.51	4 767.63	3 218.44	2 696.42	—	—	—	—	19 702.91	13 466.62	—	—

表 4 熟地黄环烯醚萜类成分结果比较（$\bar{x} \pm s$）

Table 4 Comparison on iridoids in process of *Rehmanniae Radix Praeparata* ($\bar{x} \pm s$)

蒸晒次数	梓醇/(μg·g^{-1})			地黄苷 A/(μg·g^{-1})			地黄苷 D/(μg·g^{-1})			益母草苷/(μg·g^{-1})		
	$\bar{x} \pm s$	t	P	$\bar{x} \pm s$	t	P	$\bar{x} \pm s$	t	P	$\bar{x} \pm s$	t	P
1	7 554.786 80±2 826.851 02	2.673	0.116	291.792 40± 64.966 45	4.491	0.046*	81.494 35±25.502 19	3.196	0.086	3 294.208 40±1 007.521 71	3.270	0.082
2	13 109.375 40± 620.221 76	21.137	0.002**	227.013 40± 3.086 17	73.558	0.000**	36.579 37±52.343 91	0.699	0.557	6 868.608 00± 708.769 74	9.691	0.010**
3	5 989.711 20±1 035.674 24	5.783	0.029*	724.628 80± 11.612 36	62.401	0.000**	72.081 11± 5.409 20	13.326	0.006**	984.939 00± 171.962 28	5.728	0.029*
4	3 263.739 60± 578.331 53	5.643	0.030*	264.974 80± 17.470 85	15.167	0.004**	79.938 21±12.090 61	6.612	0.022*	488.488 20± 304.548 46	1.604	0.250
5	626.649 10± 770.225 73	0.814	0.501	338.813 80±115.199 51	2.941	0.099	21.910 63±26.416 88	0.829	0.494	2 340.532 20± 34.579 01	67.687	0.000**
6	1 099.223 40± 574.881 75	1.912	0.196	492.894 20± 5.631 82	87.520	0.000**	42.920 55±37.321 19	1.150	0.369	2 142.967 40± 43.506 92	49.256	0.000**
7	546.517 40± 368.387 58	1.484	0.276	848.740 80± 38.986 85	21.770	0.002**	170.656 82±29.578 83	5.770	0.029*	1 962.712 60± 21.120 51	92.929	0.000**
8	102.054 50± 93.698 58	10.972	0.008**	754.758 40± 17.331 36	43.549	0.001**	317.963 49±64.852 41	4.903	0.039*	–	–	–
9	1 293.630 80± 45.832 84	28.225	0.001**	319.365 00± 31.541 68	10.125	0.010**	258.440 08±81.982 82	3.153	0.088	–	–	–

$^{*}P$＜0.05 $^{**}P$＜0.01，下同

$^{*}P$ <0.05 $^{**}P$ <0.01, same as below

表 5 熟地黄毛蕊花糖苷及 5-HMF 成分结果比较

Table 5 Comparison on acteoside and 5-HMF in process of *Rehmanniae Radix Praeparata*

蒸晒次数	毛蕊花糖苷/(μg·g^{-1})			5-HMF/(μg·g^{-1})		
	$\bar{x} \pm s$	t	P	$\bar{x} \pm s$	t	P
1	75.788± 4.334	17.486	0.003**	41.582± 0.552	75.322	0.000**
2	76.204±22.853	3.334	0.079	106.133± 1.226	86.562	0.000**
3	8.932± 1.453	6.149	0.025*	92.782± 4.212	22.030	0.002**
4	34.568± 7.477	4.624	0.044*	36.462± 1.404	25.968	0.001**
5	50.240±12.305	4.083	0.055	30.648± 17.723	1.729	0.226
6	130.103± 6.586	19.754	0.003**	69.628± 0.513	135.769	0.000**
7	32.744± 4.270	7.669	0.017*	316.258± 28.019	11.287	0.008**
8	59.152± 1.801	32.836	0.001**	541.798±169.003	3.206	0.085
9	56.724± 0.779	72.833	0.000**	574.012± 45.091	12.730	0.006**

蒸晒次数的增加而减小，且清蒸法与酒蒸法制得熟地黄中毛蕊花糖苷的量降低速度以第 3 次蒸晒为分界；然伴随蒸晒次数的增加，酒蒸法毛蕊花糖苷的量降低速度小于清蒸法，且于第 6 次蒸晒时毛蕊花糖苷的量存在波动。

就 5-HMF 而言，伴随蒸晒次数的增加，清蒸

表 6 熟地黄糖类成分结果比较

Table 6 Comparison on sugers in process of *Rehmanniae Radix Praeparata*

蒸晒次数	果糖/(μg·g⁻¹) $\bar{x}\pm s$	t	P	葡萄糖/(μg·g⁻¹) $\bar{x}\pm s$	t	P	蔗糖/(μg·g⁻¹) $\bar{x}\pm s$	t	P
1	98.521± 4.312	22.847	0.002**	367.546±111.001	3.311	0.080	951.056± 34.057	27.925	0.001**
2	1 175.948±134.817	8.723	0.013*	103.773± 29.818	3.480	0.740	45.515±168.789	0.270	0.813
3	292.035±148.326	1.969	0.188	304.077±128.310	2.370	0.141	35.279± 33.797	1.044	0.406
4	1 595.807±105.940	15.063	0.004**	492.236± 82.252	5.984	0.027*	921.493±137.765	6.689	0.022*
5	33.741± 89.513	0.377	0.742	204.876± 12.105	16.925	0.003**	168.618± 26.732	6.308	0.024*
6	15.400±156.198	0.099	0.930	343.962±110.874	3.102	0.090	360.007± 26.567	13.551	0.005**
7	179.256±112.766	1.590	0.253	10.620± 17.141	0.620	0.599	238.031± 10.306	23.096	0.002**
8	291.366±143.682	2.028	0.180	411.492± 29.905	13.760	0.005**	–	–	–
9	280.878±167.340	1.678	0.235	522.016±101.193	2.159	0.036*	–	–	–

蒸晒次数	棉子糖/(μg·g⁻¹) $\bar{x}\pm s$	t	P	甘露三糖/(μg·g⁻¹) $\bar{x}\pm s$	t	P	水苏糖/(μg·g⁻¹) $\bar{x}\pm s$	t	P
1	578.529±167.786	3.448	0.075	1 383.913± 425.121	3.255	0.083	2 445.478± 526.623	4.644	0.043*
2	921.624± 61.583	14.966	0.004**	3 819.858± 399.254	9.567	0.011*	2 115.166± 825.269	2.563	0.124
3	333.326± 54.385	6.129	0.026*	2 017.612± 364.923	5.556	0.031	1 276.448± 397.005	3.215	0.085
4	637.960±121.416	5.254	0.034*	3 119.077± 64.248	48.547	0.000**	5 908.892±1 219.119	4.847	0.040*
5	85.607± 26.333	3.251	0.083	765.765± 785.236	0.975	0.432	380.062± 140.010	2.715	0.113
6	–	–	–	2 836.453± 728.698	3.892	0.060	848.173± 115.483	7.345	0.018*
7	–	–	–	3 824.703± 519.336	7.365	0.018*	1 072.825± 30.700	34.946	0.001**
8	–	–	–	4 583.773± 511.501	8.961	0.012*	–	–	–
9	–	–	–	6 236.293±1 111.797	5.609	0.030*	–	–	–

法与酒蒸法制得熟地黄中 5-HMF 的量均呈增加趋势，且存在显著差异；在第 4 至第 5 次蒸晒前，清蒸法熟地黄的 5-HMF 的量略大于酒蒸法，然第 6 次蒸晒后，酒蒸法所得熟地黄的 5-HMF 的量增幅较大，明显大于清蒸法。

就糖类成分而言，伴随蒸晒次数的增加，清蒸法与酒蒸法制得熟地黄中水苏糖、蔗糖、棉子糖的量均降低，果糖、葡萄糖、甘露三糖的量均升高。清蒸法与酒蒸法经不同蒸晒次数所制备的熟地黄蔗糖、水苏糖及甘露三糖整体差异性显著。具体表现为酒蒸法第 4 次蒸晒存在波动，即水苏糖、蔗糖、棉子糖的量大于相应清蒸法，果糖、葡萄糖、甘露三糖的量小于相应清蒸法。

3 讨论

在古法熟地黄九蒸九晒的炮制过程中，发生明显的美拉德反应；且炮制辅料黄酒对炮制品的质量存在显著影响，并清蒸法与酒蒸法均以第 3、4、6 次蒸晒所得熟地黄的相关物质的量呈较大的波动性，其确切机制尚需进一步研究。

参考文献

[1] 尚志钧. 《本草经集注》概述 [J]. 安徽中医学院学报, 1983(2): 51-52.

[2] 中国药典 [S]. 二部. 2010.

[3] 林维启. 《金匮要略方论》用药特点 [J]. 甘肃中医学院学报, 1987, 3: 8-9.

[4] 秦昆明, 束雅春, 曹 岗, 等. 中药炮制研究的思路与方法—以地黄的炮制研究为例 [J]. 中草药, 2013, 44(11): 1363-1371.

[5] 卢鹏伟. 地黄的化学成分和炮制的比较研究 [D]. 郑州: 河南大学, 2008.

[6] 郭艳霞. 美拉德反应与地黄炮制机理的关系研究 [D]. 济南: 山东大学, 2012.

[7] 张丽萍, 李 军, 张振凌, 等. 熟地黄清蒸和九蒸九晒炮制品中还原糖含量测定 [J]. 河南中医学院学报, 2005, 20(4): 22-23.

[8] 刘 明, 李更生, 王慧森, 等. 地黄九蒸九晒炮制过程中益母草苷的含量测定及其动态变化 [J]. 中国药学杂志, 2009, 44(9): 658-660.
